# Integrating lipid-related composite indices and explainable machine learning for coronary heart disease risk assessment

**DOI:** 10.3389/fpubh.2026.1849932

**Published:** 2026-06-17

**Authors:** Yanchao Liu, Xuli Chen, Yuelin Hu, Kaiyu Shi, Wenwen Xiao, Chenchen Ang

**Affiliations:** 1 Department of Electrocardiology, The Second Affiliated Hospital of Wannan Medical University, Wuhu, China; 2 Anhui College of Traditional Chinese Medicine, Wuhu, China; 3 Eastern Theater Command Centers for Disease Control and Prevention, Nanjing, China; 4 Jinghu District Hospital of Wuhu City, Wuhu, China

**Keywords:** CHD, lipid, machine learning, risk assessment, SHAP

## Abstract

**Background:**

Composite indices integrating inflammation and lipid metabolism have emerged as promising markers for coronary heart disease (CHD), yet their comparative performance and discriminative ability for identifying CHD status remain incompletely understood.

**Methods:**

In this hospital-based study, 270 patients were enrolled, including 99 with CHD and 171 without CHD. Exposures included C-reactive protein (CRP) and composite indices (TG/HDL, LDL/HDL, AIP, CRP/HDL, and CRP/TG). Logistic regression, restricted cubic spline (RCS), and subgroup analyses were used to evaluate associations with CHD. Machine learning models were developed using significant predictors, and model performance was assessed by AUC, calibration, and decision curve analysis. SHapley Additive exPlanations (SHAP) were applied to interpret model outputs.

**Results:**

After multivariable adjustment, TG/HDL (OR = 2.74, 95% CI: 1.10–7.10), LDL/HDL (OR = 3.01, 95% CI: 1.21–7.81), and AIP (OR = 6.59, 95% CI: 1.61–28.51) were associated with increased odds of CHD, whereas CRP and CRP-based indices were not. RCS analyses indicated no significant nonlinearity, suggesting monotonic associations. Subgroup analyses showed generally consistent results across key strata. In classification modeling, ensemble tree-based methods performed best, with random forest and XGBoost achieving the highest discrimination ability (AUC = 0.748). SHAP analysis identified age and lipid-related composite indices as the primary contributors to CHD classification.

**Conclusion:**

Lipid-related composite indices, particularly TG/HDL, LDL/HDL, and AIP, are robust markers associated with CHD status and can be effectively integrated into machine learning models for individualized CHD classification.

## 1 Introduction

Coronary heart disease (CHD) represents a leading category of cardiovascular disorders globally and is a primary clinical manifestation of ischemic heart disease (1). According to the World Health Organization, approximately 19.8 million deaths were attributed to cardiovascular diseases in 2022, accounting for nearly 32% of all deaths worldwide, with over 75% occurring in low- and middle-income countries (2). In addition, estimates from the Global Burden of Disease study indicate that cardiovascular diseases were responsible for around 19.4 million deaths and approximately 438 million disability-adjusted life years (DALYs) in 2021, highlighting the considerable burden of mortality and disability linked to CHD and related conditions (3, 4). From a public health perspective, sustained and standardized epidemiological surveillance of CHD is essential for identifying high-risk populations, tracking temporal trends, and informing prevention strategies and health-resource allocation (5, 6).

The development of CHD results from the interplay between non-modifiable determinants, such as age, sex, and genetic predisposition, and modifiable risk factors, including smoking, hypertension, dyslipidemia, diabetes, obesity, and insufficient physical activity (7, 8). Beyond conventional lipid measurements, inflammation has emerged as an important component of atherogenesis (9). High-sensitivity C-reactive protein (hs-CRP) is regarded as a risk enhancer in selected individuals without ASCVD, and the 2024 ESC guideline for chronic coronary syndromes recommends hs-CRP assessment in patients with suspected CAD (10). However, although CRP is consistently associated with CHD/CVD risk, its causal role and incremental value in routine prediction remain debated. In parallel, composite lipid-related indices have attracted increasing attention (11). Evidence suggests that the LDL/HDL ratio may provide superior predictive ability for coronary atherosclerosis compared with either lipid parameter alone. Similarly, the TG/HDL ratio and the atherogenic index of plasma (AIP) have been linked to CHD/CAD risk and clinical outcomes in multiple studies and meta-analyses (12, 13). At the same time, evidence is not fully consistent: some studies report that TG/HDL provides little additional prognostic information beyond HDL alone, and recent meta-analytic work on AIP still calls for further studies to determine optimal cut-offs and predictive performance across populations (14). Composite inflammation-lipid markers, such as hs-CRP/HDL, have also shown prognostic value, but their role in CHD risk stratification remains insufficiently validated (15, 16) These considerations highlight the need to further evaluate CRP, TG/HDL, LDL/HDL, AIP, CRP/HDL, and CRP-triglyceride-based indices in relation to CHD.

The present study uses real-world inpatient data from a hospital-based population to comprehensively investigate the associations between inflammatory and lipid-related composite indices and CHD. Unlike prior studies that have often focused on a single biomarker or a single analytic framework, we applied a multi-method approach, including logistic regression, restricted cubic spline analysis, subgroup analyses, machine learning models, and SHAP-based interpretation, to evaluate the adjusted associations and potential nonlinear patterns of these indices in relation to CHD. By combining conventional epidemiological analysis with machine learning and explainable AI, this study provides a more comprehensive assessment of inflammatory and lipid composite markers in CHD risk evaluation and may offer preliminary evidence for future screening and risk stratification studies.

## 2 Method

### 2.1 Study population

Eligible participants in this study were consecutively recruited from patients admitted to The Second Affiliated Hospital of Wannan Medical University between August 2023 and December 2025. Enrollment was conducted at the time of hospitalization. Written informed consent was obtained from each participant prior to inclusion. The study protocol received approval from the Ethics Committee of The Second Affiliated Hospital of Wannan Medical University (approval no. WYEFYLS2026007).

### 2.2 Exposures

In the present study, the primary exposures comprised CRP along with several composite indices integrating inflammatory and lipid parameters, including the CRP-to-high-density lipoprotein cholesterol ratio (CRP/HDL), triglyceride-to–high-density lipoprotein cholesterol ratio (TG/HDL), low-density lipoprotein cholesterol-to-high-density lipoprotein cholesterol ratio (LDL/HDL), the atherogenic index of plasma (AIP), and the CRP-to-triglyceride ratio (CRP/TG). These indicators were calculated based on standard laboratory tests collected at the time of hospital admission. AIP was defined as the base-10 logarithmic transformation of the TG/HDL ratio. All exposure variables were treated as continuous measures in the analysis.

### 2.3 Outcomes

The primary outcome of this study was CHD. CHD was defined according to documented clinical diagnoses made by attending cardiologists during hospitalization, based on comprehensive clinical evaluation including symptoms, electrocardiographic findings, laboratory examinations, imaging results when available, and previous history of coronary artery disease.

### 2.4 Covariates

The covariates included in this study were age, sex, smoking status, drinking status, and diabetes. These variables were collected from the baseline admission records and medical charts. Age was analyzed as a continuous variable, whereas sex, smoking status, drinking status, and diabetes were treated as categorical variables. Smoking status was classified as current/past smoking versus never smoking, and drinking status was classified similarly according to the available clinical records. Diabetes was defined based on the documented medical history or physician diagnosis at admission. These covariates were selected because they are well-recognized factors associated with cardiovascular risk and may potentially confound the relationship between the exposures and CHD.

### 2.5 Statistical analysis

Continuous variables were summarized as mean ± standard deviation (SD) for normally distributed data or as median with interquartile range (IQR) for skewed distributions. Normality was assessed before comparison. Between-group differences in normally distributed continuous variables were assessed using the independent-samples Student’s t-test, while the Mann–Whitney U test was used for non-normally distributed continuous variables. Categorical variables were described using frequencies and percentages and compared using the chi-square test.

The relationships between exposures and CHD were initially examined using logistic regression analyses. Exposures were modeled both as continuous variables and as categorical variables based on quartile classification (Q1-Q4), with the lowest quartile (Q1) defined as the reference group. Effect estimates were reported as odds ratios (ORs) with corresponding 95% confidence intervals (CIs). To evaluate the stability of the associations, a sequence of regression models with progressive adjustment for potential confounders was constructed: Model 1 included no covariate adjustment; Model 2 controlled for age and sex; and Model 3 further adjusted for smoking status, alcohol consumption, and diabetes.

Potential non-linear dose–response relationships between exposures and CHD were explored using restricted cubic spline (RCS) functions with three knots positioned at selected percentiles of the exposure distribution. Restricted cubic spline analyses with three knots placed at the 10th, 50th, and 90th percentiles of the exposure variable were used to assess potential nonlinear associations. Non-linearity was tested by comparing a model containing only the linear term with a model incorporating spline terms via a likelihood ratio test.

For classification modeling, variables that demonstrated significant associations in multivariable logistic regression were selected as candidate features. The dataset was randomly partitioned into a training set (70%) and a testing set (30%) using a fixed random seed. Categorical variables were transformed into dummy variables based on the training dataset, and the same encoding scheme was subsequently applied to the testing dataset. A total of ten machine learning algorithms were implemented, including Random Forest (RF), Extreme Gradient Boosting (XGBoost), Neural Network (NNet), Elastic Net (EN), Naïve Bayes (NB), k-Nearest Neighbors (kNN), Logistic Regression (LR), Gradient Boosting Machine (GBM), Support Vector Machine (SVM), and Classification and Regression Tree (CART). Model development was conducted using 5-fold cross-validation within the training set. Hyperparameter optimization was performed using grid-search-based tuning procedures to maximize the cross-validated area under the receiver operating characteristic curve (AUC). For each algorithm, predefined candidate parameter ranges were evaluated, and the parameter combination yielding the best average cross-validation performance was selected as the final model. Calibration performance was evaluated through calibration plots comparing classified and observed CHD status probabilities. In addition, decision curve analysis (DCA) was performed to examine the potential clinical applicability of the models across a range of threshold probabilities. Among the algorithms tested, the XGBoost model demonstrated superior overall classification performance and was therefore selected for further interpretation. To enhance model transparency, SHapley Additive exPlanations (SHAP) were employed to quantify the contribution of each predictor. Visualization techniques, including SHAP summary plots, dependence plots, and force plots, were used to illustrate both global and individual-level feature effects on CHD classification.

All statistical analyses were carried out using R software (version 4.5.2). Statistical significance was defined as a two-tailed *p* value < 0.05.

## 3 Results

### 3.1 Basic characteristics of patients

A total of 270 patients were included in the analysis, of whom 171 were classified as having no CHD and 99 as having CHD. As shown in Table 1, patients with CHD were significantly older than those without CHD. No significant differences were observed in sex distribution, smoking status, or drinking status between the two groups. In contrast, diabetes was more prevalent in the CHD group. In addition, several lipid parameters and inflammatory/lipid-related composite indices, including TG, HDL, LDL, CRP, TG/HDL, LDL/HDL, AIP, CRP/HDL, and CRP/TG, differed significantly between the CHD and non-CHD groups, suggesting distinct cardiometabolic profiles in patients with CHD.

**Table 1:** Basic characteristics of participants.

Characteristics	Overall	No CHD	CHD	*p* value
N	270	171	99	
Age [mean (SD)]	68.94 (12.46)	65.33 (12.11)	75.18 (10.46)	<0.001
Sex (%)				
Male	125 (46.3)	77 (45.0)	48 (48.5)	0.673
Female	145 (53.7)	94 (55.0)	51 (51.5)	
Smoke (%)				
Yes	25 (9.3)	16 (9.4)	9 (9.1)	1
No	245 (90.7)	155 (90.6)	90 (90.9)	
Drinking (%)				
Yes	24 (8.9)	16 (9.4)	8 (8.1)	0.894
No	246 (91.1)	155 (90.6)	91 (91.9)	
Diabetes (%)				
Yes	42 (15.6)	15 (8.8)	27 (27.3)	<0.001
No	228 (84.4)	156 (91.2)	72 (72.7)	
TG [mg/dL, median (IQR)]	1.08 (0.79, 1.70)	1.19 (0.86, 1.73)	0.98 (0.71, 1.44)	0.007
HDL [mg/dL, median (IQR)]	2.14 (1.67, 2.87)	2.49 (1.88, 3.18)	1.73 (1.42, 2.17)	<0.001
LDL [mg/dL, median (IQR)]	1.05 (0.88, 1.23)	1.07 (0.93, 1.25)	1.02 (0.83, 1.17)	0.032
CRP [mg/L, median (IQR)]	4.90 (1.40, 17.50)	3.70 (0.95, 14.90)	6.00 (2.45, 26.90)	0.025
TG/HDL [median (IQR)]	0.54 (0.39, 0.71)	0.52 (0.36, 0.69)	0.56 (0.43, 0.77)	0.024
LDL/HDL [median (IQR)]	0.48 (0.36, 0.65)	0.45 (0.34, 0.56)	0.58 (0.43, 0.75)	<0.001
AIP [median (IQR)]	−0.27 (−0.41, −0.15)	−0.29 (−0.44, −0.16)	−0.25 (−0.37, −0.11)	0.024
CRP/HDL [median (IQR)]	2.06 (0.54, 9.14)	1.46 (0.46, 6.94)	4.07 (1.45, 12.37)	0.001
CRP/TG [median (IQR)]	4.52 (0.97, 17.44)	3.33 (0.77, 13.62)	5.70 (1.80, 29.89)	0.004

SD, standard deviation; TG, triglycerides; HDL, high-density lipoprotein; LDL, low-density lipoprotein; AIP, atherogenic index of plasma; CRP, C-reactive protein; IQR, interquartile range.

### 3.2 Associations between exposure and CHD

The associations between the investigated exposures and CHD are summarized in Table 2. In the unadjusted model, CRP, TG/HDL, LDL/HDL, AIP, and CRP/HDL were all significantly related to CHD. After adjustment for age and sex in Model 2, however, the associations for CRP and CRP-based composite indices were no longer statistically significant, whereas TG/HDL, LDL/HDL, and AIP remained significant. In the fully adjusted model, these three lipid-related indices were associated with higher odds of CHD. Specifically, TG/HDL was linked to a 2.74-fold increase in CHD odds (OR = 2.74, 95% CI: 1.10–7.10, *p* = 0.034), LDL/HDL to a 3.01-fold increase (OR = 3.01, 95% CI: 1.21–7.81, *p* = 0.019), and AIP to a 6.59-fold increase (OR = 6.59, 95% CI: 1.61–28.51, *p* = 0.010). By contrast, CRP, CRP/HDL, and CRP/TG showed no significant associations with CHD.

**Table 2:** Association between composite inflammatory/lipid markers and CHD.

Model	CRP	*p* value	TG/HDL	*p* value	LDL/HDL	*p* value	AIP	*p* value	CRP/HDL	*p* value	CRP/TG	*p* value
	OR (95% CI)		OR (95% CI)		OR (95% CI)		OR (95% CI)		OR (95% CI)		OR (95% CI)	
Model 1	1.01 (1.00, 1.02)	0.030	2.29 (1.04, 5.17)	0.041	3.62 (1.53, 9.46)	0.005	4.68 (1.34, 16.92)	0.017	1.02 (1.00, 1.03)	0.018	1.01 (1.00, 1.01)	0.119
Model 2	1.01 (0.99, 1.02)	0.170	2.94 (1.21, 7.48)	0.021	3.01 (1.22, 7.86)	0.019	7.45 (1.88, 31.41)	0.005	1.01 (1.00, 1.03)	0.104	1.00 (0.99, 1.01)	0.623
Model 3	1.00 (0.99, 1.01)	0.466	2.74 (1.10, 7.10)	0.034	3.01 (1.21, 7.81)	0.019	6.59 (1.61, 28.51)	0.010	1.01 (0.99, 1.02)	0.323	1.000 (0.99, 1.01)	0.904

Model 1: not adjusted. Model 2: adjusted for age and sex. Model 3: further adjusted for smoking status, alcohol consumption, and diabetes.

The quartile-based analyses were broadly consistent with these findings (Supplementary Table S1). Compared with participants in the lowest quartile, those in higher TG/HDL quartiles had greater odds of CHD (Q2: OR = 2.84, 95% CI: 1.26–6.63; Q3: OR = 3.09, 95% CI: 1.35–7.32; Q4: OR = 2.87, 95% CI: 1.27–6.67; P for trend = 0.018). A similar dose–response pattern was observed for LDL/HDL, with a significant increasing trend across quartiles (Q4 vs. Q1: OR = 3.22, 95% CI: 1.43–7.52; P for trend = 0.001), and AIP also demonstrated a significant trend. In contrast, no meaningful trend was detected for CRP, CRP/HDL, or CRP/TG. The variance inflation factor (VIF) values for all variables were <5 (Supplementary Table S2), indicating no significant multicollinearity among the covariates.

### 3.3 Nonlinear relationship

**Figure 1:**
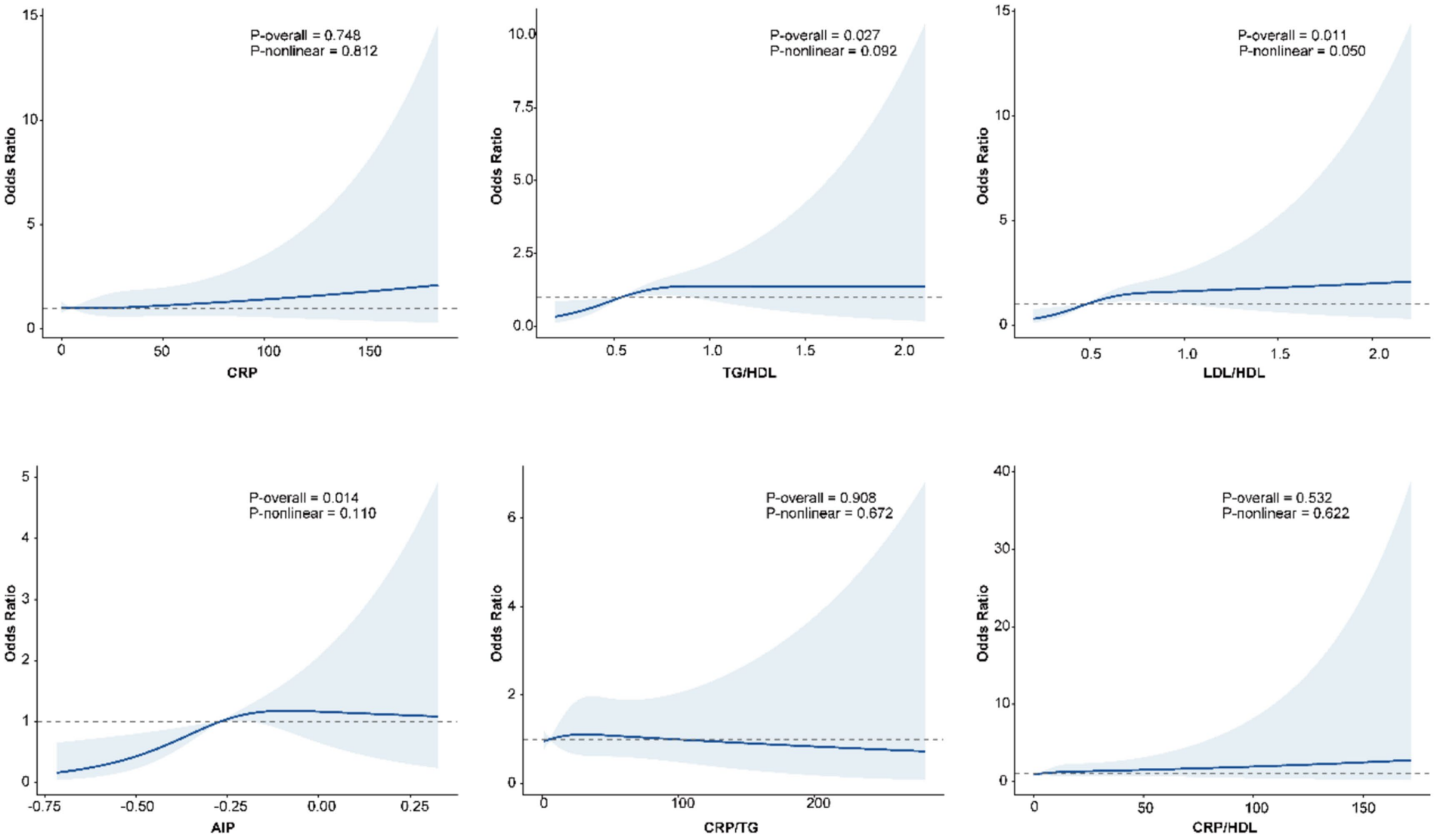
Non-linear association between a composite inflammation/lipid index and CHD.

Restricted cubic spline (RCS) analyses were conducted to explore the potential nonlinear relationships between exposures and CHD (Figure 1). Overall, no significant evidence of nonlinearity was observed for the investigated indices (all P for non-linearity > 0.05). Consistent with the logistic regression results, TG/HDL, LDL/HDL, and AIP showed a monotonic increasing association with the risk of CHD across their distributions. In contrast, CRP, CRP/HDL, and CRP/TG did not exhibit clear dose–response patterns.

### 3.4 Subgroup analysis

**Figure 2:**
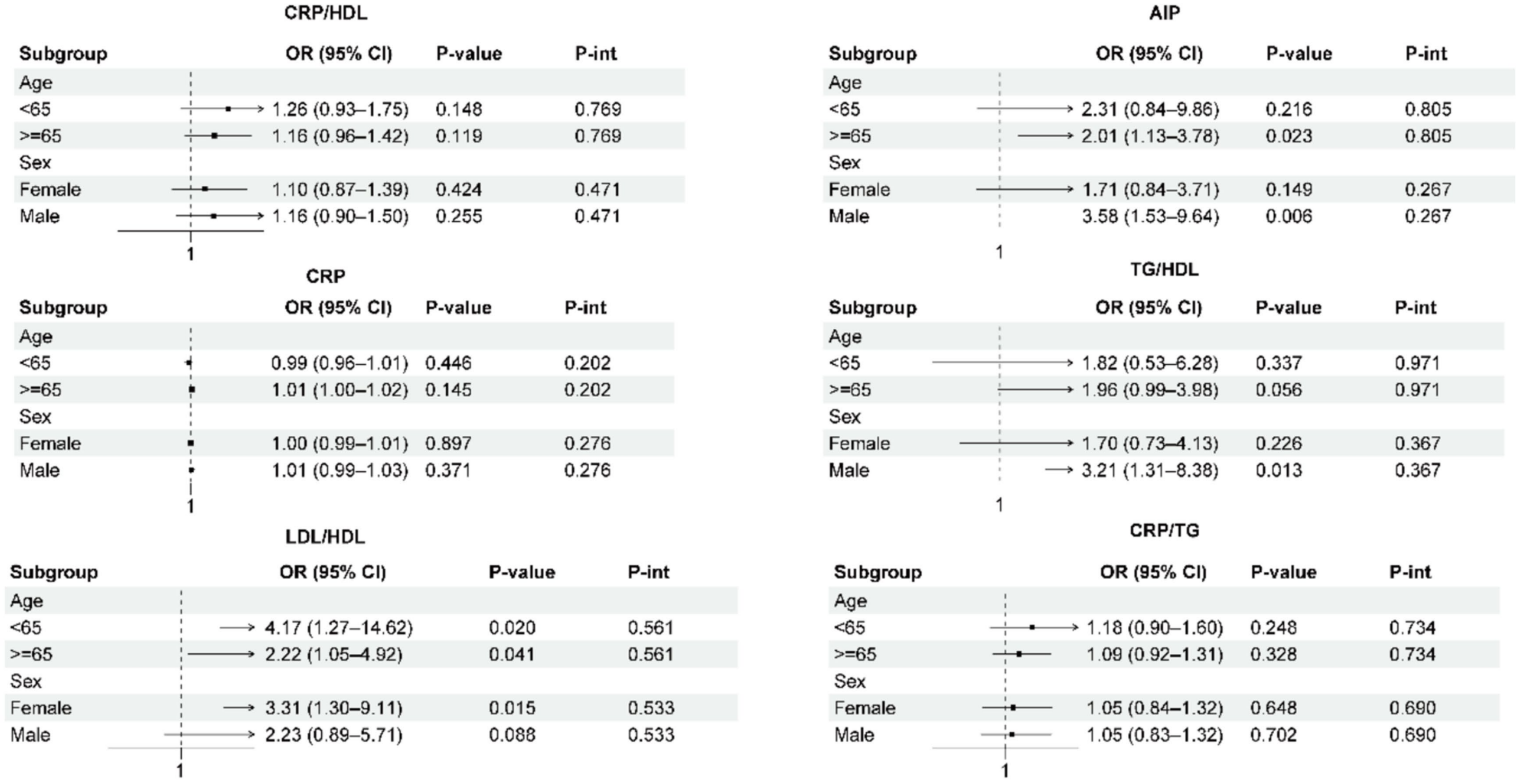
Subgroup analysis of the inflammation/lipid composite index and CHD.

Subgroup analyses assessed associations between exposures and CHD across age, sex, smoking, alcohol use, and diabetes (Figure 2 and Supplementary Tables S3–S8). Specifically, TG/HDL showed generally consistent positive associations, notably in males (OR = 3.21, 95% CI: 1.31–8.38), non-smokers (OR = 2.51, 95% CI: 1.32–4.93), non-drinkers (OR = 2.33, 95% CI: 1.24–4.48), and participants without diabetes (OR = 2.13, 95% CI: 1.09–4.29). LDL/HDL was positively associated with CHD in both age groups (<65: OR = 4.17, 95% CI: 1.27–14.62; ≥65: OR = 2.22, 95% CI: 1.05–4.92) and in females (OR = 3.31, 95% CI: 1.30–9.11), with a significant interaction by drinking (*p* = 0.012; OR in drinkers = 304.81, 95% CI: 5.43–488637.64) and a borderline interaction by smoking (*p* = 0.085). AIP showed consistent positive associations across subgroups, particularly in older participants (≥65: OR = 2.01, 95% CI: 1.13–3.78), males (OR = 3.58, 95% CI: 1.53–9.64), non-smokers (OR = 2.56, 95% CI: 1.46–4.78), non-drinkers (OR = 2.41, 95% CI: 1.39–4.41), and those without diabetes (OR = 2.51, 95% CI: 1.38–4.95). CRP and CRP-based composite indices were mostly non-significant, except for a smoking interaction for CRP (*p* = 0.049).

### 3.5 Development and validation of the CHD classification model

**Figure 3:**
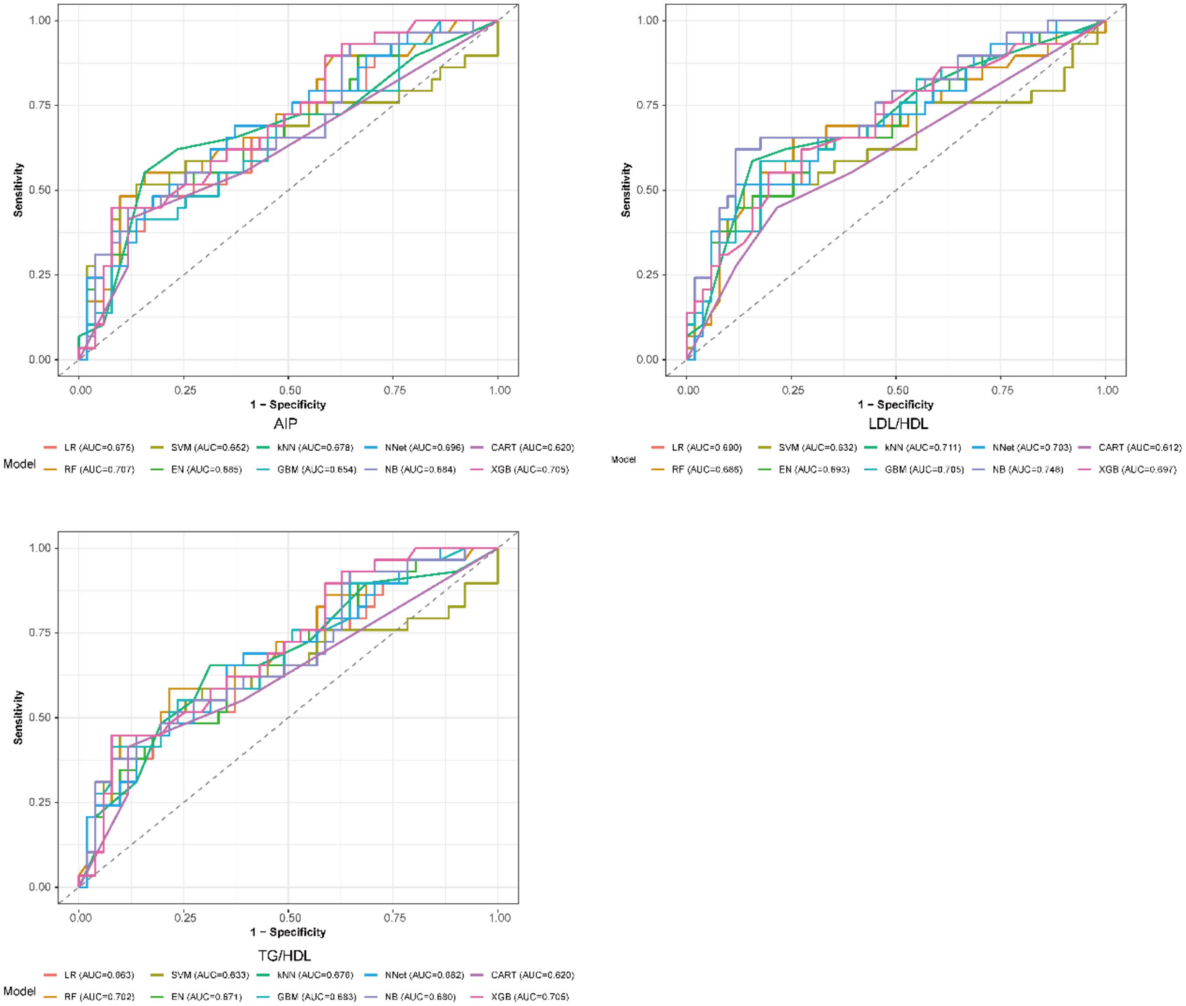
ROC curves of machine learning models for prediction composite lipid indices and CHD.

**Figure 4:**
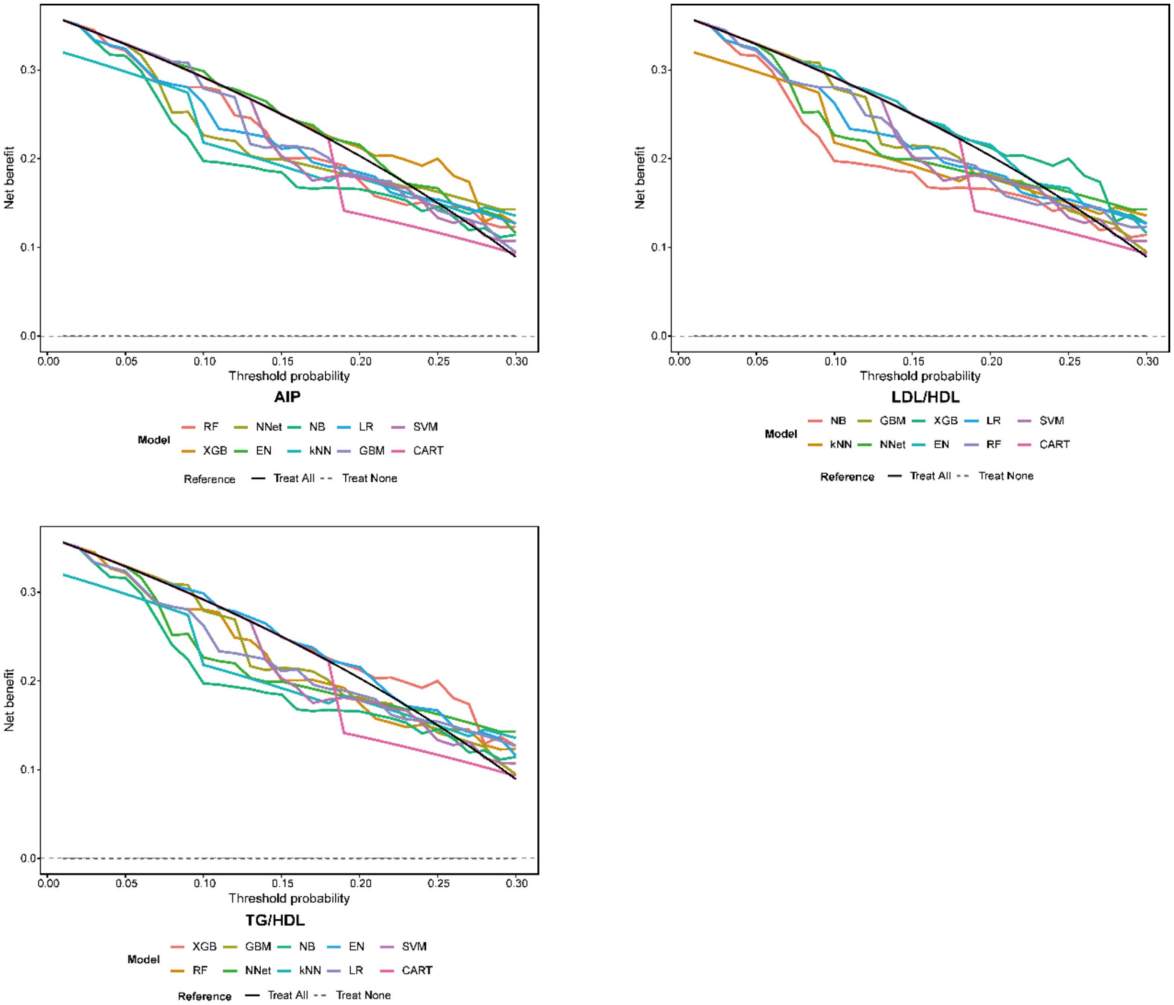
Decision curve analysis of machine learning models for prediction composite lipid indices and CHD.

**Figure 5:**
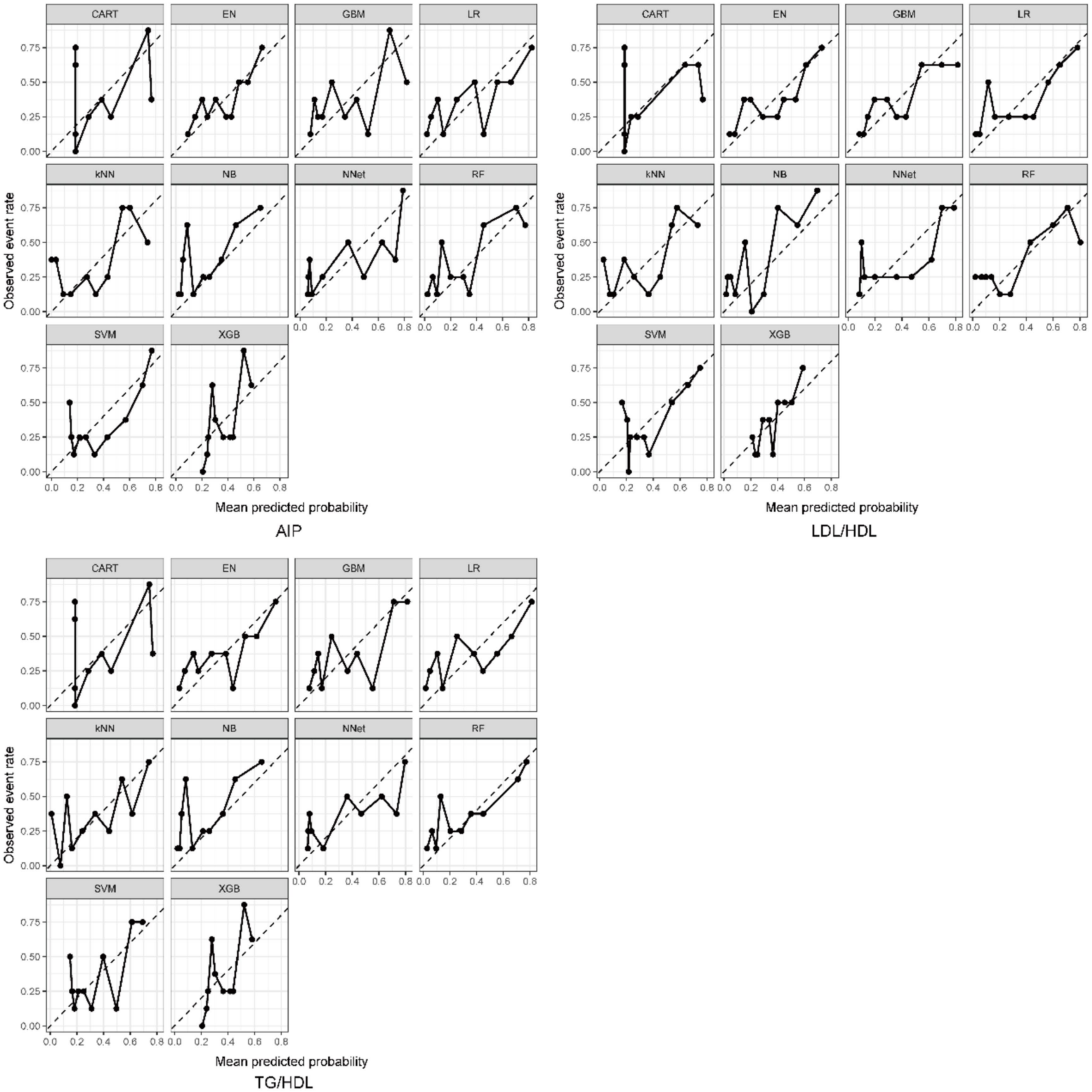
Calibration curves of machine learning models for prediction composite lipid indices and CHD.

Given that AIP, LDL/HDL, and TG/HDL showed significant associations with CHD in previous analyses, we constructed machine learning-based models for CHD status classification using these indices (Figures 3–5; Supplementary Tables S9, S10). Among AIP-based models, RF and XGB achieved the highest discrimination (AUC = 0.707 and 0.705, respectively) with RF showing balanced sensitivity (0.552) and specificity (0.843), while XGB exhibited higher specificity (0.922) but lower sensitivity (0.448). For LDL/HDL, NB performed best (AUC = 0.748, accuracy = 0.788, sensitivity = 0.621, specificity = 0.882). TG/HDL models also demonstrated superior performance with ensemble tree-based methods (RF: AUC = 0.702; XGB: AUC = 0.705). ROC curves confirmed robust discrimination of the top-performing models, DCA indicated greater potential clinical utility for RF and XGB across a range of thresholds, and calibration curves showed good agreement between estimated and observed CHD status probabilities. These results indicate that RF and XGB can effectively integrate lipid indices for CHD status classification with satisfactory discrimination and calibration performance.

### 3.6 SHAP interpretive analysis

**Figure 6:**
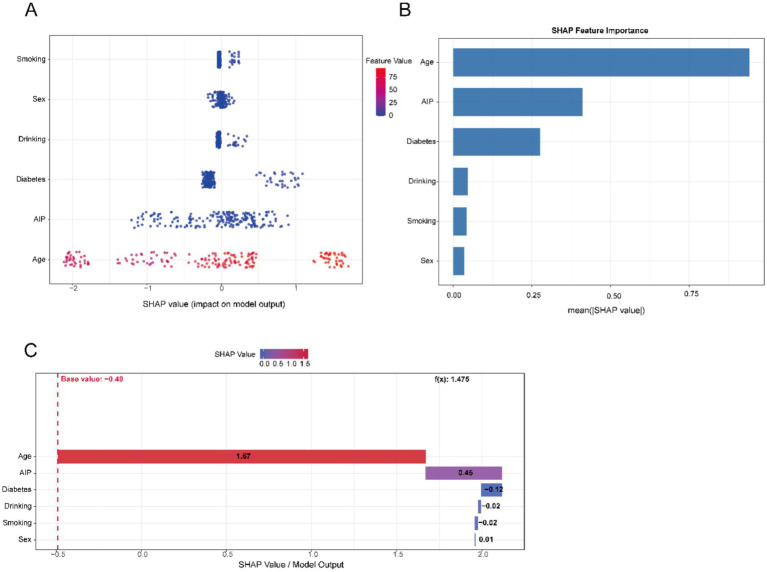
SHAP analysis of AIP in the XGBoost model for CHD prediction. **(A)** SHAP summary plot. **(B)** SHAP dependence plot. **(C)** SHAP force plot.

**Figure 7:**
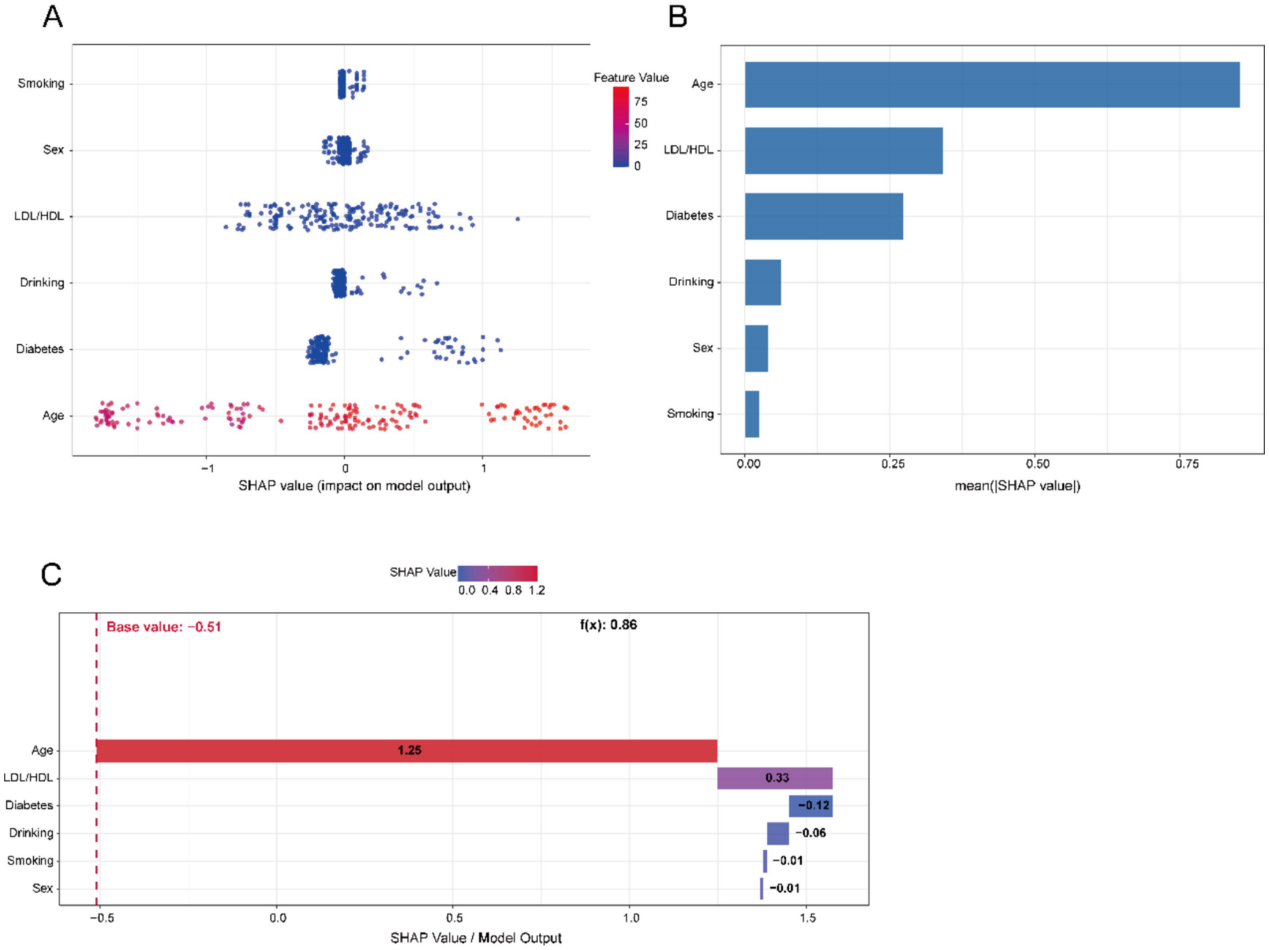
SHAP analysis of LDL/HDL in the XGBoost model for CHD prediction. **(A)** SHAP summary plot. **(B)** SHAP dependence plot. **(C)** SHAP force plot.

**Figure 8:**
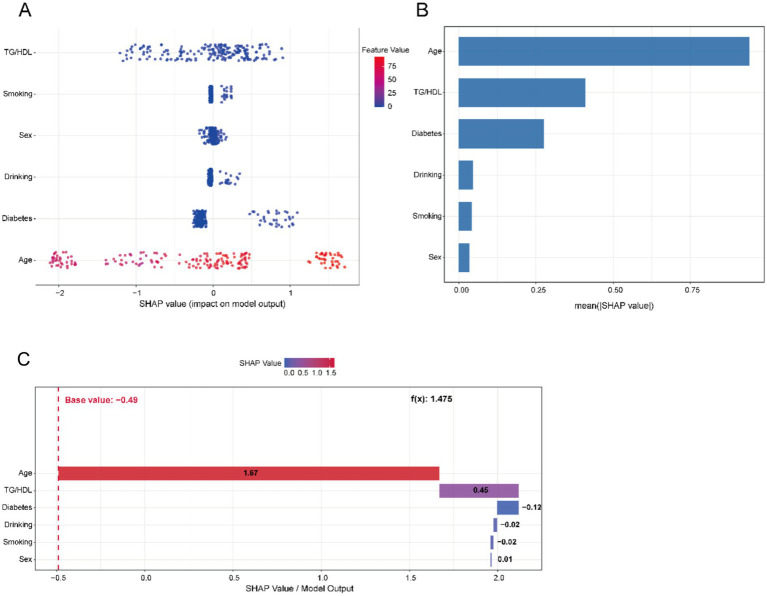
SHAP analysis of TG/HDL in the XGBoost model for CHD prediction. **(A)** SHAP summary plot. **(B)** SHAP dependence plot. **(C)** SHAP force plot.

Because XGBoost showed the best overall performance, SHAP was used to interpret the XGBoost-based models for AIP, LDL/HDL, and TG/HDL (Figures 6–8). In all three SHAP summary and feature-importance plots, age emerged as the most influential feature, followed by the corresponding composite lipid index (AIP, LDL/HDL, or TG/HDL), while diabetes contributed moderately and smoking, drinking, and sex had relatively small effects. The dependence patterns suggested that higher age and higher values of the composite indices generally shifted the model toward a higher estimated probability of CHD classification. The force plots further illustrated that, in individual classifications, age made the largest positive contribution to the final model output, with the composite index providing additional contribution to CHD classification, whereas the remaining covariates produced only minor shifts. Collectively, these SHAP results indicated that the XGBoost models were driven mainly by age and the lipid-related composite indices, and they provided an interpretable basis for CHD status classification.

## 4 Discussion

In this hospital-based real-world study of 270 patients, we found that patients with CHD were older and had a more adverse cardiometabolic profile than those without CHD, with significant differences in several lipid parameters and inflammation/lipid-related composite indices. After multivariable adjustment, TG/HDL, LDL/HDL, and AIP showed associated with higher odds of CHD, whereas CRP and CRP-based composite indices were not consistently associated with disease status. Restricted cubic spline analyses suggested no evidence of nonlinearity, indicating a largely monotonic exposure-risk pattern for the significant lipid composites. These associations were generally consistent across clinically relevant subgroups. In addition, machine learning models demonstrated moderate discriminatory performance, with RF and XGBoost showing the best overall classification performance, and SHAP analysis further identified age and the corresponding composite lipid index as the dominant contributors to model outputs. Collectively, these findings suggest that lipid-related composite indices, particularly TG/HDL, LDL/HDL, and AIP, may serve as practical markers for CHD status assessment, while also supporting their potential value in individualized CHD classification models.

Our findings showed that TG/HDL, LDL/HDL, and AIP remained associated with CHD after multivariable adjustment, which is broadly consistent with prior epidemiological evidence. TG/HDL has been widely considered a simple surrogate marker of insulin resistance and atherogenic dyslipidemia. Previous evidence supports its prognostic value, including a meta-analysis of 13 cohort studies involving 207,515 participants showing a significantly higher risk of cardiovascular events in the highest versus lowest TG/HDL category, together with a clear dose–response pattern, and a Korean cohort reporting a stepwise increase in incident ischemic heart disease risk across TG/HDL quartiles (17, 18). These findings suggest that TG/HDL reflects not only metabolic derangement but also meaningful future CHD risk. LDL/HDL also showed a robust association with CHD in our study. By integrating atherogenic LDL and protective HDL into a single metric, this ratio may better capture overall lipid imbalance than either component alone. Prior meta-analytic and angiographic studies have similarly shown that LDL/HDL is higher in CHD populations, performs better than isolated lipid measures in discriminating coronary disease, and is related to coronary stenosis severity (19). In the same vein, AIP, as a logarithmic transformation of TG/HDL, may more sensitively reflect triglyceride-rich lipoprotein excess, low HDL, remnant lipoprotein abnormalities, and insulin resistance-related atherogenicity (20). Recent systematic reviews, meta-analyses, and cohort studies have consistently linked higher AIP with CAD occurrence, disease severity, and adverse outcomes, supporting its value as an integrated marker of cardiometabolic risk (21). By contrast, CRP and CRP-based composite indices were not associated with CHD in the fully adjusted models. Although CRP is a well-established marker of systemic inflammation and has been linked to cardiovascular risk in large prospective studies, its incremental value beyond conventional cardiometabolic factors appears limited and remains debated (9). In a relatively small hospital-based sample with substantial adjustment for age, sex, and metabolic covariates, attenuation of the CRP effect is plausible. Taking together, these results support the clinical relevance of lipid-related composite indices, particularly TG/HDL, LDL/HDL, and AIP, as practical markers for CHD risk assessment.

In subgroup analyses, the associations of TG/HDL, LDL/HDL, and AIP with CHD were generally consistent across major demographic and clinical strata, underscoring the robustness of these lipid-related composite indices. Notably, stronger or more stable associations were observed in males, which is broadly consistent with prior epidemiological evidence suggesting that these indices may better capture atherogenic dyslipidemia in populations with lower baseline metabolic disturbance (22, 23). Previous studies have demonstrated that TG/HDL and AIP, as markers of insulin resistance and triglyceride-rich lipoprotein metabolism, exhibit relatively stable associations with cardiovascular risk across sex and age groups, while LDL/HDL has been consistently linked to both the presence and severity of atherosclerosis in diverse populations (24–26). Overall, these findings suggest that TG/HDL, LDL/HDL, and AIP are relatively stable and generalizable markers of CHD risk, with limited effect modification across common subgroups, while also highlighting the need for cautious interpretation of subgroup-specific estimates.

In the present study, RF and XGBoost showed the highest discrimination among the models tested, but overall performance remained moderate. This is consistent with prior studies showing that ensemble methods can be useful in cardiovascular prediction tasks, although performance varies across datasets and study settings (27, 28). This finding is consistent with previous studies demonstrating the superior predictive ability of tree-based ensemble models in cardiovascular risk prediction (29). For example, prior research has shown that RF and XGBoost generally outperform traditional models and simpler algorithms in terms of discrimination, calibration, and clinical utility, largely due to their ability to capture complex nonlinear relationships and feature interactions (30–33). In particular, XGBoost has been repeatedly reported to achieve high accuracy, stability, and resistance to overfitting, with strong generalizability across datasets and favorable net clinical benefit in decision curve analyses (34, 35). Our findings, showing moderate but robust discrimination and good calibration, further support the applicability of these models in real-world clinical settings, even when constructed using a limited number of composite lipid features. Importantly, beyond model performance, our study incorporated SHAP (SHapley Additive exPlanations) to enhance interpretability, addressing a key limitation of machine learning models often regarded as “black boxes.” In line with prior studies, our SHAP analysis also suggested that age and the lipid-related composite indices were the main contributors to model output (36–38). Moreover, SHAP dependence and force plots in our study revealed clear directionality of effects, with increasing values of TG/HDL, LDL/HDL, and AIP contributing positively to CHD risk, which is biologically plausible and consistent with their roles in atherogenic dyslipidemia (39–41). Taken together, these findings highlight several important implications. First, lipid-related composite indices can be effectively integrated into machine learning frameworks to achieve reliable CHD classification with acceptable discrimination and clinical applicability. Second, the use of SHAP provides a transparent and interpretable framework that bridges the gap between model performance and clinical interpretability, thereby enhancing the potential for real-world applications. Third, machine learning models based on routinely available biomarkers may provide an adjunctive aid for CHD classification rather than a stand-alone diagnostic tool. Overall, our results suggest that the combined use of machine learning and explainable AI may provide a useful and interpretable framework for CHD risk classification and support further exploration in future studies. However, we acknowledge that internal validation cannot fully eliminate the possibility of optimistic performance estimates in a small sample.

This study has several notable strengths and innovations. First, it integrates multiple inflammation- and lipid-related composite indices, within a unified analytical framework, allowing for a comprehensive comparison of their relative contributions to CHD risk. Unlike prior studies that typically focus on single biomarkers, our approach provides a more holistic evaluation of the interplay between lipid metabolism and inflammation. Second, we combined traditional epidemiological methods with advanced analytical techniques, including restricted cubic spline analysis, subgroup analysis, and machine learning models, enabling a more nuanced assessment of both linear and potential nonlinear associations, as well as population heterogeneity. Third, by incorporating a broad range of machine learning algorithms and systematically evaluating their performance using discrimination, calibration, and decision curve analysis, we identified robust and clinically useful classification models based on simple, routinely available biomarkers. Importantly, we further enhanced model interpretability by applying SHAP. Finally, the use of real-world hospital-based data strengthens the clinical relevance and applicability of our findings, supporting the potential utility of these composite indices and machine learning–based classification models in routine CHD status assessment.

This study also has several limitations. First, the relatively small sample size from a single center may limit statistical power, particularly in subgroup analyses, and may reduce the generalizability of the results to broader populations. Second, although we adjusted for several key confounders, residual confounding from unmeasured variables, such as dietary factors, physical activity, medication use (e.g., lipid-lowering or anti-inflammatory drugs), and socioeconomic status, cannot be excluded. Third, exposure variables were measured at a single time point at admission, which may not reflect long-term metabolic or inflammatory status and could lead to misclassification. Fourth, due to the cross-sectional and hospital-based design of this study, causal relationships between lipid-related indices and CHD cannot be inferred. Furthermore, the developed models reflect discriminatory performance within the study population rather than true prospective risk prediction. Fifth, because CHD diagnosis was based on real-world clinical records rather than uniform protocol-driven diagnostic criteria or mandatory coronary angiography for all participants, some degree of diagnostic heterogeneity and potential misclassification cannot be excluded. In addition, although SHAP analysis in this study was used solely to improve interpretability of the best-performing machine learning model and should not be interpreted as mechanistic evidence. Given the modest sample size and the possibility of instability in fitted models, SHAP-derived feature importance estimates should be considered exploratory and require confirmation in larger independent datasets. The machine learning models demonstrated reasonable performance, external validation in independent cohorts is needed to confirm their robustness and clinical utility. Finally, the present study did not compare the proposed models with established cardiovascular risk prediction tools such as Framingham Risk Score, SCORE2, or ASCVD score, the incremental clinical value of the lipid composite–based models cannot be determined. Future studies should perform direct head-to-head comparisons with conventional risk prediction systems and standard lipid-based regression models in larger independent cohorts.

## 5 Conclusion

Lipid-related composite indices, particularly TG/HDL, LDL/HDL, and AIP, emerged as robust and important markers of CHD risk in this study and demonstrated consistent associations across multiple analytical approaches. These indices, derived from routinely available clinical parameters, can be effectively integrated into machine learning models to achieve reliable and individualized risk assessment. Importantly, the combination of traditional epidemiological methods with explainable machine learning techniques, such as SHAP, provides a transparent and interpretable framework that enhances both model performance and clinical applicability. Collectively, our findings highlight the potential of lipid-related composite markers as practical tools for CHD risk stratification and support the integration of explainable artificial intelligence into clinical decision-making. Further validation in larger, multi-center prospective studies is warranted to confirm their generalizability and utility in diverse populations.

## Data Availability

The original contributions presented in the study are included in the article/Supplementary material, further inquiries can be directed to the corresponding authors.
