# Supplementary material for: Integrating lipid-related composite indices and explainable machine learning for coronary heart disease risk assessment
Source: Front Public Health. 2026 Jun 17;14:1849932. doi: 10.3389/fpubh.2026.1849932 (PMC13319073; doi:10.3389/fpubh.2026.1849932)
Supplement: Supplementary file 1 [file Supplementary_file_1.DOCX]

**Integrating Lipid-Related Composite Indices and Explainable Machine Learning for Coronary Heart Disease Risk Assessment**

Table S1. Association between composite inflammatory/lipid markers and CHD by quaritile.

|  | Q1 | Q2 | Q3 | Q4 | P-trend |
| --- | --- | --- | --- | --- | --- |
| CRP | Ref | 0.668 (0.290, 1.513) | 0.850 (0.385, 1.870) | 0.952 (0.422, 2.141) | 0.919 |
| TG/HDL | Ref | 2.844 (1.259, 6.629) | 3.092 (1.351, 7.322) | 2.870 (1.273, 6.667) | 0.018 |
| LDL/HDL | Ref | 0.894 (0.369, 2.166) | 2.399 (1.044, 5.675) | 3.220 (1.426, 7.524) | 0.001 |
| AIP | Ref | 2.844 (1.259, 6.629) | 3.092 (1.351, 7.322) | 2.870 (1.273, 6.667) | 0.018 |
| CRP/HDL | Ref | 0.804 (0.349, 1.840) | 1.137 (0.506, 2.567) | 1.148 (0.505, 2.618) | 0.546 |
| CRP/TG | Ref | 0.931 (0.408, 2.118) | 1.328 (0.595, 2.980) | 0.974 (0.426, 2.228) | 0.835 |

Table S2. Variance inflation factor (VIF) analysis for multivariable logistic regression models

| Variable | VIF |
| --- | --- |
| tg_hdl | 1.025 |
| age | 1.112 |
| sex | 1.115 |
| smoke | 1.832 |
| drink | 1.841 |
| diabetes | 1.006 |
|  |  |
| ldl_hdl | 1.022 |
| age | 1.081 |
| sex | 1.127 |
| smoke | 1.883 |
| drink | 1.914 |
| diabetes | 1.006 |
|  |  |
| aip | 1.030 |
| age | 1.118 |
| sex | 1.117 |
| smoke | 1.838 |
| drink | 1.845 |
| diabetes | 1.006 |
|  |  |
| crp_hdl | 1.073 |
| age | 1.087 |
| sex | 1.112 |
| smoke | 1.839 |
| drink | 1.879 |
| diabetes | 1.053 |
|  |  |
| crp_tg | 1.076 |
| age | 1.098 |
| sex | 1.111 |
| smoke | 1.852 |
| drink | 1.888 |
| diabetes | 1.051 |

Table S3. Subgroup analysis of the association between CRP and CHD.

| Subgroup | OR (95% CI) | P-value | P-interaction |
| --- | --- | --- | --- |
| Age |  |  |  |
| <65 | 0.99 (0.96–1.01) | 0.446 | 0.202 |
| >=65 | 1.01 (1.00–1.02) | 0.145 |  |
| Sex |  |  |  |
| Female | 1.00 (0.99–1.01) | 0.897 | 0.276 |
| Male | 1.01 (0.99–1.03) | 0.371 |  |

Table S4. Subgroup analysis of the association between TG/HDL and CHD.

| Subgroup | OR (95% CI) | P-value | P-interaction |
| --- | --- | --- | --- |
| Age |  |  |  |
| <65 | 1.82 (0.53–6.28) | 0.337 | 0.971 |
| >=65 | 1.96 (0.99–3.98) | 0.056 |  |
| Sex |  |  |  |
| Female | 1.70 (0.73–4.13) | 0.226 | 0.367 |
| Male | 3.21 (1.31–8.38) | 0.013 |  |

Table S5. Subgroup analysis of the association between LDL/HDL and CHD.

| Subgroup | OR (95% CI) | P-value | P-interaction |
| --- | --- | --- | --- |
| Age |  |  |  |
| <65 | 4.17 (1.27–14.62) | 0.020 | 0.561 |
| >=65 | 2.22 (1.05–4.92) | 0.041 |  |
| Sex |  |  |  |
| Female | 3.31 (1.30–9.11) | 0.015 | 0.533 |
| Male | 2.23 (0.89–5.71) | 0.088 |  |

Table S6. Subgroup analysis of the association between AIP and CHD.

| Subgroup | OR (95% CI) | P-value | P-interaction |
| --- | --- | --- | --- |
| Age |  |  |  |
| <65 | 2.31 (0.84–9.86) | 0.216 | 0.805 |
| >=65 | 2.01 (1.13–3.78) | 0.023 |  |
| Sex |  |  |  |
| Female | 1.71 (0.84–3.71) | 0.149 | 0.267 |
| Male | 3.58 (1.53–9.64) | 0.006 |  |

Table S7. Subgroup analysis of the association between CRP/HDL and CHD.

| Subgroup | OR (95% CI) | P-value | P-interaction |
| --- | --- | --- | --- |
| Age |  |  |  |
| <65 | 1.26 (0.93–1.75) | 0.147903 | 0.768599 |
| >=65 | 1.16 (0.96–1.42) | 0.118856 | 0.768599 |
| Sex |  |  |  |
| Female | 1.10 (0.87–1.39) | 0.424464 | 0.470652 |
| Male | 1.16 (0.90–1.50) | 0.254509 | 0.470652 |

Table S8. Subgroup analysis of the association between CPR/TG and CHD.

| Subgroup | OR (95% CI) | P-value | P-interaction |
| --- | --- | --- | --- |
| Age |  |  |  |
| <65 | 1.18 (0.90–1.60) | 0.248 | 0.734 |
| >=65 | 1.09 (0.92–1.31) | 0.328 |  |
| Sex |  |  |  |
| Female | 1.05 (0.84–1.32) | 0.648 | 0.690 |
| Male | 1.05 (0.83–1.32) | 0.702 |  |

Table S9. Metrics of machine learning models in predicting AIP and CHD.

| Model | AUC (95% CI) | Accuracy | Sensitivity | Specificity | PPV | NPV | F1 | BrierScore | Calibration_Intercept | Calibration_Slope |
| --- | --- | --- | --- | --- | --- | --- | --- | --- | --- | --- |
| RF | 0.707 (0.585-0.828) | 0.738 | 0.552 | 0.843 | 0.667 | 0.768 | 0.604 | 0.206 | -0.003 | 0.561 |
| XGB | 0.705 (0.587-0.822) | 0.750 | 0.448 | 0.922 | 0.765 | 0.746 | 0.565 | 0.207 | 0.203 | 1.363 |
| NNet | 0.696 (0.575-0.817) | 0.650 | 0.690 | 0.627 | 0.513 | 0.780 | 0.588 | 0.226 | -0.205 | 0.430 |
| EN | 0.685 (0.562-0.808) | 0.725 | 0.448 | 0.882 | 0.684 | 0.738 | 0.542 | 0.210 | -0.055 | 0.792 |
| NB | 0.684 (0.559-0.808) | 0.725 | 0.448 | 0.882 | 0.684 | 0.738 | 0.542 | 0.228 | 0.207 | 0.498 |
| kNN | 0.678 (0.548-0.809) | 0.738 | 0.552 | 0.843 | 0.667 | 0.768 | 0.604 | 0.219 | -0.420 | 0.027 |
| GLM | 0.675 (0.551-0.799) | 0.700 | 0.483 | 0.824 | 0.609 | 0.737 | 0.538 | 0.226 | -0.213 | 0.408 |
| GBM | 0.654 (0.527-0.781) | 0.725 | 0.379 | 0.922 | 0.733 | 0.723 | 0.500 | 0.226 | -0.251 | 0.461 |
| SVM | 0.652 (0.510-0.795) | 0.738 | 0.517 | 0.863 | 0.682 | 0.759 | 0.588 | 0.208 | -0.215 | 0.665 |
| CART | 0.620 (0.497-0.744) | 0.713 | 0.414 | 0.882 | 0.667 | 0.726 | 0.511 | 0.221 | -0.235 | 0.536 |

Table S10. Metrics of machine learning models in predicting LDL/HDL and CHD.

| Model | AUC (95% CI) | Accuracy | Sensitivity | Specificity | PPV | NPV | F1 | BrierScore | Calibration_Intercept | Calibration_Slope |
| --- | --- | --- | --- | --- | --- | --- | --- | --- | --- | --- |
| NB | 0.748 (0.631-0.865) | 0.788 | 0.621 | 0.882 | 0.750 | 0.804 | 0.679 | 0.196 | 0.365 | 0.653 |
| kNN | 0.711 (0.588-0.834) | 0.750 | 0.586 | 0.843 | 0.680 | 0.782 | 0.630 | 0.207 | -0.461 | 0.044 |
| GBM | 0.705 (0.584-0.827) | 0.738 | 0.586 | 0.824 | 0.654 | 0.778 | 0.618 | 0.208 | -0.199 | 0.629 |
| NNet | 0.703 (0.580-0.825) | 0.750 | 0.517 | 0.882 | 0.714 | 0.763 | 0.600 | 0.214 | -0.181 | 0.529 |
| XGB | 0.697 (0.573-0.821) | 0.713 | 0.552 | 0.804 | 0.615 | 0.759 | 0.582 | 0.204 | 0.250 | 1.492 |
| EN | 0.693 (0.570-0.816) | 0.725 | 0.448 | 0.882 | 0.684 | 0.738 | 0.542 | 0.215 | -0.152 | 0.556 |
| GLM | 0.690 (0.567-0.814) | 0.713 | 0.517 | 0.824 | 0.625 | 0.750 | 0.566 | 0.219 | -0.192 | 0.447 |
| RF | 0.686 (0.557-0.815) | 0.713 | 0.655 | 0.745 | 0.594 | 0.792 | 0.623 | 0.212 | -0.168 | 0.443 |
| SVM | 0.632 (0.489-0.775) | 0.738 | 0.517 | 0.863 | 0.682 | 0.759 | 0.588 | 0.207 | -0.170 | 0.767 |
| CART | 0.612 (0.489-0.735) | 0.663 | 0.448 | 0.784 | 0.542 | 0.714 | 0.491 | 0.233 | -0.290 | 0.447 |

Table S11. Metrics of machine learning models in predicting TG/HDL and CHD.

| Model | AUC (95% CI) | Accuracy | Sensitivity | Specificity | PPV | NPV | F1 | BrierScore | Calibration_Intercept | Calibration_Slope |
| --- | --- | --- | --- | --- | --- | --- | --- | --- | --- | --- |
| XGB | 0.705 (0.587-0.822) | 0.750 | 0.448 | 0.922 | 0.765 | 0.746 | 0.565 | 0.207 | 0.203 | 1.363 |
| RF | 0.702 (0.580-0.824) | 0.713 | 0.586 | 0.784 | 0.607 | 0.769 | 0.596 | 0.206 | -0.009 | 0.564 |
| GBM | 0.683 (0.558-0.807) | 0.738 | 0.414 | 0.922 | 0.750 | 0.734 | 0.533 | 0.218 | -0.234 | 0.534 |
| NNet | 0.682 (0.559-0.805) | 0.713 | 0.448 | 0.863 | 0.650 | 0.733 | 0.531 | 0.230 | -0.228 | 0.412 |
| NB | 0.680 (0.555-0.805) | 0.713 | 0.448 | 0.863 | 0.650 | 0.733 | 0.531 | 0.230 | 0.175 | 0.475 |
| kNN | 0.676 (0.551-0.802) | 0.675 | 0.655 | 0.686 | 0.543 | 0.778 | 0.594 | 0.219 | -0.495 | 0.020 |
| EN | 0.671 (0.547-0.795) | 0.688 | 0.483 | 0.804 | 0.583 | 0.732 | 0.528 | 0.222 | -0.182 | 0.486 |
| GLM | 0.663 (0.538-0.788) | 0.675 | 0.483 | 0.784 | 0.560 | 0.727 | 0.519 | 0.231 | -0.225 | 0.384 |
| SVM | 0.633 (0.489-0.777) | 0.738 | 0.448 | 0.902 | 0.722 | 0.742 | 0.553 | 0.212 | -0.102 | 0.695 |
| CART | 0.620 (0.497-0.744) | 0.713 | 0.414 | 0.882 | 0.667 | 0.726 | 0.511 | 0.221 | -0.235 | 0.536 |
